# Case Report: No Evidence of Intracranial Fluid Shifts in an Astronaut Following an Aborted Launch

**DOI:** 10.3389/fneur.2021.774805

**Published:** 2021-12-09

**Authors:** Heather R. McGregor, Kathleen E. Hupfeld, Ofer Pasternak, Scott J. Wood, Ajitkumar P. Mulavara, Jacob J. Bloomberg, T. Nick Hague, Rachael D. Seidler

**Affiliations:** ^1^Department of Applied Physiology and Kinesiology, University of Florida, Gainesville, FL, United States; ^2^Department of Psychiatry and Radiology, Brigham and Women's Hospital, Harvard Medical School, Boston, MA, United States; ^3^NASA Johnson Space Center, Houston, TX, United States; ^4^KBR, Houston, TX, United States; ^5^NASA Johnson Space Center, Houston, TX, United States; ^6^NASA Astronaut Corps, Houston, TX, United States; ^7^Norman Fixel Institute for Neurological Diseases, University of Florida, Gainesville, FL, United States

**Keywords:** spaceflight, launch abort, ventricular volume, free water (FW), microgravity, hypergravity

## Abstract

Spaceflight induces lasting enlargement of the brain's ventricles as well as intracranial fluid shifts. These intracranial fluid shifts have been attributed to prolonged microgravity exposure, however, the potential effects of hypergravity exposure during launch and landing have yet to be elucidated. Here we describe a case report of a Crewmember who experienced an Aborted Launch (“CAL”). CAL's launch and landing experience was dissociated from prolonged microgravity exposure. Using MRI, we show that hypergravity exposure during the aborted launch did not induce lasting ventricular enlargement or intracranial fluid shifts resembling those previously reported with spaceflight. This case study therefore rules out hypergravity during launch and landing as a contributing factor to previously reported long-lasting intracranial fluid changes following spaceflight.

## Introduction

Spaceflight induces long-lasting intracranial fluid changes. Ventricular expansion has been widely reported, ranging 11–25% from pre- to post-flight (1–10). Spaceflight also induces redistribution of free water (FW) within the cranium. FW refers to extracellular water molecules that do not experience flow and whose diffusion is not restricted by their surroundings (11). Following spaceflight, FW decreases at the top of the brain and increases around the base of the cerebrum (2, 6, 8).

These intracranial fluid changes have been attributed to prolonged microgravity exposure (1–3, 6, 8, 10). However, spaceflight also encompasses ~4.5 g hypergravity during launch and landing. Since MRI scans are acquired preflight and post-flight, it is unclear whether post-flight intracranial fluid changes are due to microgravity exposure *per se*, hypergravity exposures from launch and landing, or both. Here we present a case study of an astronaut whose experience of launch and landing was dissociated from prolonged microgravity exposure. We leveraged these data to determine whether launch and landing contribute to previously-reported fluid shifts with spaceflight (1, 2, 10).

## Case Description

The Russian Soyuz MS-10 launched from the Baikonur Cosmodrome in Kazakhstan. Approximately 2 min after liftoff, an anomaly occurred during booster separation, causing one of the boosters to strike and puncture the central core of the rocket. Crewmembers in the capsule were shaken laterally (±g_x_) and vertically (±g_z_). A launch abort was automatically triggered and thrusters fired to separate the spacecraft from the rocket. Crewmembers were abruptly thrust back into their seats, experiencing ~1 g in the anteroposterior direction (–g_y_). Once fuel was expended, the crew experienced ~3 min of microgravity as the capsule began its ballistic descent. The capsule's descent was steeper than normal, exposing crewmembers to g-forces peaking at 6.7 g in the posteroanterior direction (+g_y_) for ~5 s. During nominal landings, peak g-forces typically reach ~4–5 g. Astronauts undergo pre-flight high-g training up to 8 g in the anteroposterior direction for 30 s on a centrifuge. The landing was nominal following parachute deployment (12), with 5–6 g of lateral forces (g_x_) as the capsule rotated and swung beneath the parachute. Crewmembers experienced g-forces in the posteroanterior direction (+g_y_) at touchdown. Subjectively, touchdown felt like a car crash at ~30–50 km/h (personal communication with CAL). The capsule tumbled on the ground, exposing the crewmembers to changes in whole-body orientation relative to Earth's gravitational vector before coming to a stop. The crewmembers were safely recovered in good condition. One of the crewmembers onboard, referred to here as CAL (Crewmember who experienced the Aborted Launch), was a participant in our prospective NASA-funded study (13). As such, we acquired T1-weighted and diffusion-weighted magnetic resonance imaging (MRI) brain scans twice before the aborted launch (Pre1-2) and once afterward (Pre3). Here, we contrast CAL's brain changes to those of a control group of 12 ground-based astronauts who underwent two MRI scans pre-flight. This unique occurrence allowed us to investigate if hypergravity exposure during launch and landing altered CAL's intracranial fluid distribution.

CAL subsequently completed a long-duration ISS mission (~200 days). We acquired post-flight MRI at several points following his return from the ISS (Post1–3). This allowed us to perform a within-subject analysis to qualitatively compare CAL's intracranial fluid changes following the aborted launch to those associated with his subsequent spaceflight.

We predicted that CAL's ventricular volumes and FW distribution would be stable from before to after the launch abort. That is, we predicted that CAL's change in ventricular and FW fractional volume would be comparable to that of the control group of ground-based astronauts. We further predicted that CAL would exhibit ventricular volume increases and FW shifts consistent with previous spaceflight studies following his subsequent ISS expedition. This would support that spaceflight-associated intracranial fluid shifts arise due to prolonged microgravity exposure.

## Methods

### Participants

Fifteen astronauts participated in our prospective study between 2014 and 2020. Due to time constraints, diffusion-weighted MRI scans were not acquired from two astronauts during the Pre1 scan session. Thus, data from these two astronauts were excluded from analyses (*n* = 13: CAL and a control group of 12 astronauts, Table 1).

**Table 1:** Astronaut demographics at baseline (Pre1).

	**CAL**	**Control Group**
	**(*n* = 1)**	**(*n* = 12)**
Sex	Male	eight males, four females
Mean age, years	42.7	47 ± 6.9
Novice/Experienced	Novice	four experienced, eight novice
ISS Mission duration, days	204	–

*CAL, Crewmember who experienced the Aborted Launch. Control group data are presented as mean ± SD. “Experienced” refers to having completed at least one prior spaceflight*.

This study was approved by the Institutional Review Boards at the University of Michigan, the University of Florida, and NASA Johnson Space Center. All astronauts provided written informed consent prior to their participation. CAL provided consent for individual data presentation.

#### Crewmember Who Experienced the Aborted Launch

CAL's Pre1 and Pre2 MRI scans occurred 143 and 101 days, respectively, prior to his first scheduled launch. CAL reported no difficulty tolerating the hypergravity induced by either the preflight ballistic training nor the ballistic reentry (14). A Pre3 MRI scan was acquired 64 days after the aborted launch. Ninety days following this, CAL was successfully launched to the ISS and completed a mission lasting ~200 days. Upon the completion of CAL's ISS mission, we acquired post-flight MRI scans 4 (Post1), 30 (Post2), and 97 (Post3) days afterwards.

#### Control Group

Two pre-flight MRI scans were acquired for each of the control astronauts. The Pre1 MRI scans occurred an average of 177.3 (±65.2 SD) days prior to launch and the Pre2 occurred 64.9 (±26.9 SD) days prior to launch.

**Figure 1:**
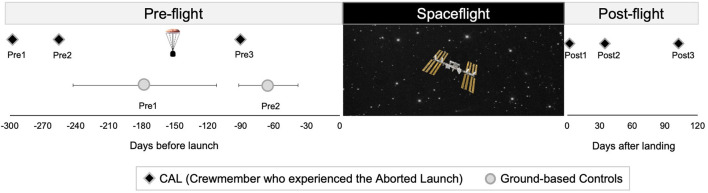
Testing Timelines. Left: timing of pre-flight MRI scans for CAL (black diamonds) and controls (circles). The capsule icon indicates the timing of the aborted launch. Control group data are presented as means, with error bars indicating SD. Right: timing of CAL's post-flight scans. CAL, Crewmember who experienced the Aborted Launch.

Astronauts in the control group then completed ISS missions lasting on average 176.2 (±43.6 SD) days. Post1 MRI scans were acquired on average 4.7 (±1.4 SD) days following their return to Earth. Post-flight MRI data collected from the control group were only used for generating a standard space region of interest (ROI) localizing brain areas of spaceflight-induced FW shifts. Figure 1 shows the testing timeline.

### Image Acquisition

All MRI scans were acquired using the same 3T Siemens Magnetom Verio MRI scanner located at University of Texas Medical Branch at Victory Lakes in Houston, TX.

T1-weighted anatomical images were collected using a magnetization-prepared rapid gradient-echo (MPRAGE) sequence: TR = 1,900 ms, TE = 2.32 ms, flip angle = 9°, FOV = 250 × 250 mm, 176 sagittal slices, slice thickness = 0.9 mm, matrix = 512 × 512, voxel size = 0.489 × 0.489 × 0.9 mm.

Diffusion-weighted MRI scans were acquired using a diffusion-weighted 2D single-shot spin-echo prepared echo-planar imaging sequence with the following parameters: TR = 11,300 ms, TE = 95 ms, flip angle = 90°, FOV = 225 × 225 mm, matrix size = 128 × 128, 40 axial slices of 2 mm thickness (zero gap), voxel size = 1.95 × 1.95 × 2 mm. Thirty non-collinear gradient directions with diffusion weighting of b = 1,000 s/mm^2^ were sampled twice. Two volumes with no diffusion weighting (b = 0 s/mm^2^) were acquired, one at the start of the run and one at the midpoint.

### Image Processing

#### T1-Weighted Image Processing

T1-weighted image processing was performed using the Computational Anatomy Toolbox (15) (CAT12.6 v.1450) for Statistical Parametric Mapping (16) Version 12 (SPM12 v.7219) implemented in Matlab R2016a, version 9.0. Each raw, native space T1 was segmented using CAT12 by employing standard preprocessing steps for longitudinal data.

#### Diffusion-Weighted Image Processing

Diffusion-weighted images were analyzed using FMRIB Software Library (17) (FSL) version 6.0.1 and a custom FW algorithm (11) implemented using Matlab R2018b.

Following visual inspection, we performed eddy current distortion correction and motion correction. Diffusion-weighted volumes were registered to the average of the two *b* = 0 volumes. Rotations applied to each volume during motion correction were also applied to corresponding b vectors. For each dMRI run, we plotted the volume-wise root mean square displacement provided by eddy. A volume was deemed an outlier if its volume-to-volume displacement exceeded 1 mm. Outlier volumes were removed from the eddy corrected image and from b value and vector matrices. dMRI runs were then skull stripped.

FW fractional volume was estimated by fitting a bi-tensor model at each voxel of the preprocessed DWI image (11). FW refers to extracellular water molecules that do not experience flow and whose diffusion is not restricted by their surroundings. FW is primarily located within the ventricles as cerebrospinal fluid (CSF) and around the brain parenchyma (11). The first tensor estimates fractional volume of FW per voxel, reflecting the proportion of water molecules with unrestricted diffusion (11). The second tensor then estimates diffusion indices of water molecules within tissue (i.e., restricted diffusion within tissue) including fractional anisotropy (FA).

### Regions of Interest

#### ROI Selection

The 64-day gap between CAL's aborted launch and Pre3 MRI precluded us from detecting transient, fast-recovering structural brain changes following the aborted launch. Spaceflight induces gray matter volume changes (2, 3, 6, 18, 19), white matter microstructure changes (8, 19), ventricular enlargement (1–5, 10), and intracranial fluid (FW, CSF) displacements (2, 6, 8, 19). There is little evidence, though, that gray and white matter changes persist 64 days post-flight. For this reason, we examined structural brain changes that have been reported to last at least 3 months following spaceflight—enlargement of the lateral and third ventricles (1, 2, 8, 10) and FW fractional volume shifts (2, 6, 8, 19). Therefore, if hypergravity exposure during launch and landing contribute to previously identified ventricular enlargement or FW shifts with spaceflight, then such changes in CAL's brain would be evident in the Pre3 MRI scans.

Ventricular and free water ROIs were defined in standard MNI space and registered to native space for volume estimations.

#### Ventricular Volume

We used CAT12 to automatically estimate the native space volumes of the left lateral, right lateral, and third ventricles using the Neuromorphometrics atlas map included in SPM12.

#### Free Water Volume

We also estimated within-subject FW fractional volume changes in native space. We first performed a between-subjects analysis to characterize pre- to post-flight FW changes in the control group of astronauts. The standard space clusters resulting from this analysis were then registered to each subject's native space image to extract the average FW fractional volume within each ROI.

We created FW ROIs in MNI standard space by analyzing the control group's FW changes from pre- to post-flight. We normalized the control group's FA maps to MNI space using a step-wise registration approach using Advanced Normalization Tools (ANTs) version 2.1.0 (20, 21). We used FA images because they provide greater anatomical detail for registration than FW maps. Since FA and FW images for a given time point were derived from a single DWI scan, the same transformations for normalizing FA images to MNI space can be applied to FW maps. As detailed elsewhere (2), our step-wise registration approach involved registering each native space FA image to MNI standard space *via* subject-specific FA templates. Briefly, for each astronaut, we registered all FA images to a subject-specific FA template using rigid, affine, and non-linear transformations. Next, we normalized each subject-specific FA template to a 1 mm resolution MNI152 standard space T1 template using rigid, affine, and Symmetric Normalization (SyN) transformations. We concatenated the transformations yielded during these registration steps into a single flow field for each subject and time point. We applied the corresponding flow field to native space FW maps, transforming each into MNI standard space.

Following MNI normalization, we quantified pre- to post-flight FW changes in each of the control astronauts by subtracting their Pre2 FW map from their Post1 FW map. Control group FW difference maps were smoothed using a 5 mm full width at half maximum Gaussian kernel. FW images were concatenated inputted into *randomize* (22), FSL's non-parametric permutation-based inference tool. Our general linear model characterized regions of FW increases and FW decreases in the control group following spaceflight, adjusting for individual differences in astronaut age, sex, mission duration, and the number of days between landing and the Post1 scan session. Analyses were performed using 4,096 (exhaustive) permutations with threshold-free cluster enhancement. Correction for multiple comparisons was implemented using a familywise error correction and contrasts were tested with two-tailed *t*-tests (*p* < 0.05). Similar to previous work using a separate group of astronauts (19), this analysis significant clusters of post-flight FW increases and FW decreases (**Figure 3A**). These clusters were binarized and used as FW ROIs.

We applied the inverse transformations to the standard space FW ROIs, transforming them to each subject's native space FA image. From each astronaut's unsmoothed FW map, we computed the average FW fractional volume across all voxels within each native space FW ROI.

### Analyses

Since this is a single individual case study, we performed qualitative analyses as opposed to statistical tests.

#### Pre-flight Slopes

**Figure 2:**
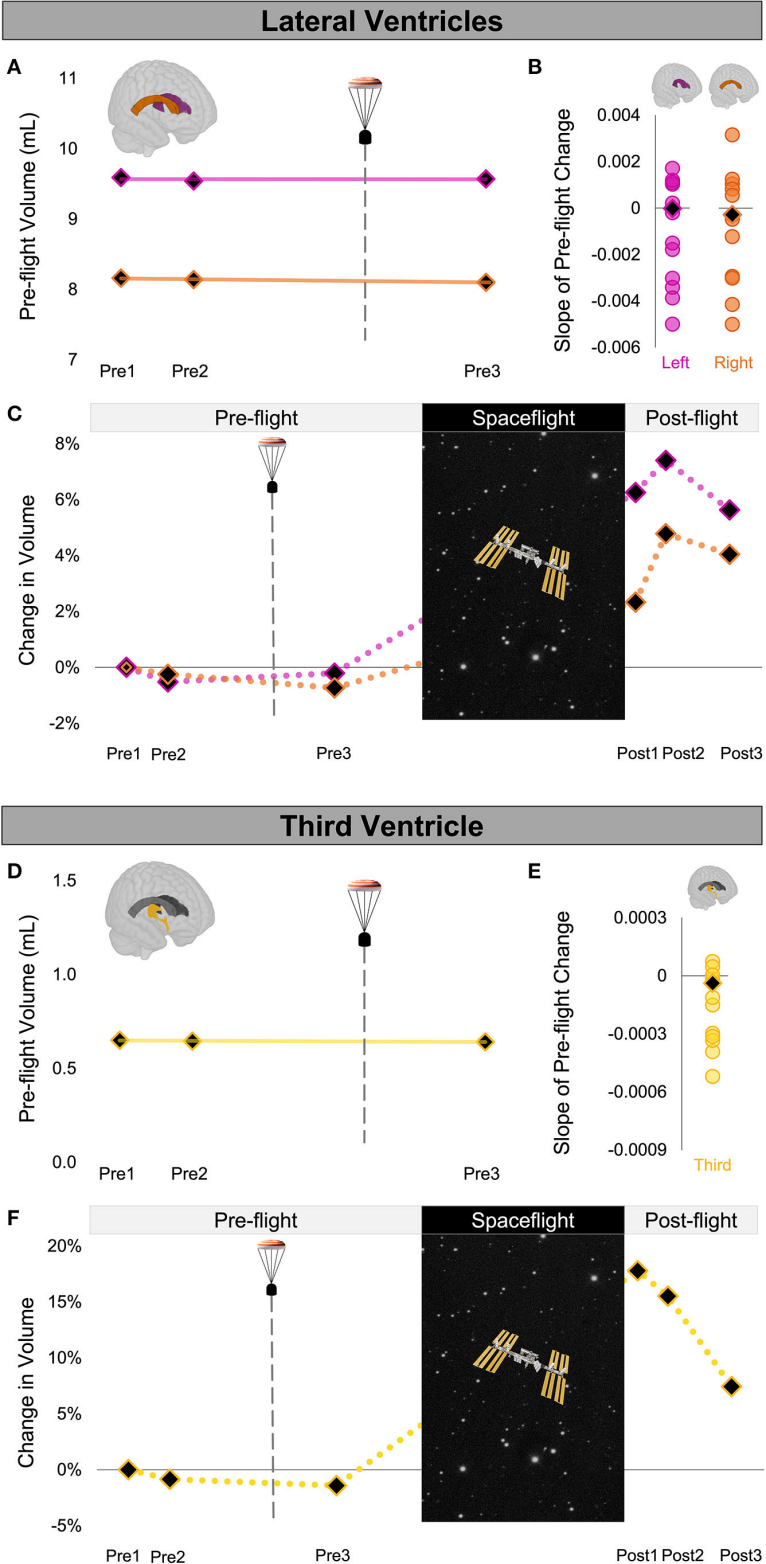
Ventricle Volume Changes Following Launch Abort and Spaceflight. **(A)** CAL's pre-flight volume changes for the left (magenta) and right (orange) lateral ventricles. The solid trend lines indicate the slopes of pre-flight ventricular volume changes. The aborted launch is indicated by the gray dashed line and capsule icon. **(B)** Slopes of CAL's pre-flight lateral ventricular volume changes (black diamond) compared to the slopes of pre-flight lateral ventricular volume changes of 12 ground-based control astronauts (circles). **(C)** CAL's lateral ventricular volume changes from before to after a 6-month expedition on the ISS. Volume changes are reflected as percent volume change relative to baseline (Pre1). **(D)** CAL's pre-flight volume changes for the third ventricle (yellow). Display conventions are as in **(A)**. **(E)** Slope of CAL's pre-flight third ventricle volume change (black diamond) compared to those of the 12 ground-based control astronauts (circles). **(F)** CAL's third ventricle volume changes from before to after a 6-month expedition on the ISS. Display conventions are as in **(C)**. CAL, Crewmember who experienced the Aborted Launch; ISS, International Space Station.

**Figure 3:**
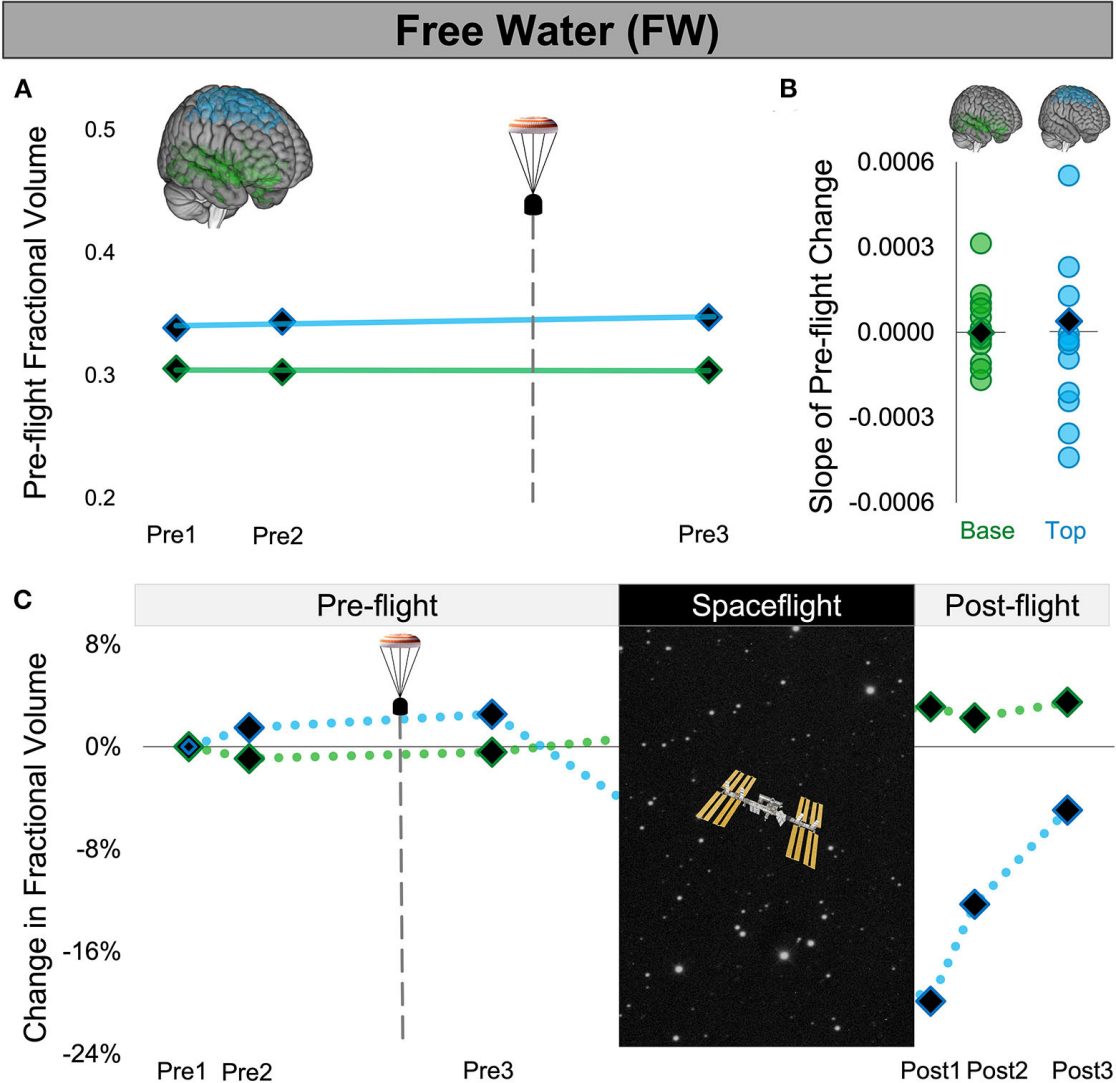
Free Water Changes Following Launch Abort and Spaceflight. **(A)** CAL's pre-flight changes in FW fractional volume at the top (blue) and base (green) of the cerebrum. The solid trend lines indicate the slopes of pre-flight ventricular volume changes. The aborted launch is indicated by the gray dashed line and capsule icon. **(B)** Slopes of CAL's pre-flight FW fractional volume changes (black diamond) compared to the slopes of pre-flight FW fractional volume changes of 12 ground-based control astronauts (circles). **(C)** CAL's FW fractional volume changes from before to after a 6-month expedition on the ISS. Volume changes are reflected as percent volume change relative to baseline (Pre1). CAL, Crewmember who experienced the Aborted Launch; FW, free water; ISS, International Space Station.

Since the number of days between pre-flight MRI scans differed between astronauts, we assessed the slope of CAL's pre-flight ROI volume changes (volume change/day). For CAL, the slope of change for each ROI was calculated across the 3 pre-flight sessions. CAL's pre-flight slopes thus reflected the rate of ROI volume change per day from before (Pre1,2) to after the aborted launch (Pre3) as shown in Figures 2A,D, 3A. For each control astronaut, the slope of change for each ROI volume was calculated across the 2 pre-flight sessions (Pre1,2). The control groups' pre-flight slopes thus reflected the rate of ROI volume changes occurring due to aging, training, and other pre-flight experiences [see (23) for a review]. We computed Z scores to quantify whether the slopes of CAL's pre-flight ROI changes were outliers relative to those of the control group.

#### Within-Subject Comparison of Launch Abort and Spaceflight Effects

For CAL only, we performed a within-subject qualitative analysis to compare ROI volume changes from pre- to post-flight. For each ROI, we computed the percent ROI volume change for each session relative to baseline (Pre1) according to the following equation:


$$\begin{array}{l} PercentChange_{i}\;=\;\frac{Volume_{i}-Volume_{Pre1}}{Volume_{Pre1}}\;x\;100\% \end{array}$$

Volume_*i*_ indicates the ROI volume at a given session, *i*. Volume_*Pre*1_ indicates the ROI volume at the baseline Pre1 session. This equation thus yields the percent change in ROI volume for a session relative to baseline as shown in Figures 2C,F, 3C and Table 2.

**Table 2:** CAL's percent change in ROI volume at each session relative to the baseline Pre1 session.

**Region of Interest**		**Percent Change of ROI Volume**					**Ratio of Spaceflight vs. Launch Abort Change**
		**Pre2**	**Pre3**	**Post1**	**Post2**	**Post3**	
Ventricle	Left lateral	−0.52%	−0.21%	6.26%	7.40%	5.63%	−26.8
	Right lateral	−0.25%	−0.74%	2.33%	4.78%	4.04%	−5.46
	Third	−0.85%	−1.42%	17.79%	15.51%	7.43%	−5.23
Free Water	Top of cerebrum	1.49%	2.54%	−19.90%	−12.31%	−4.96%	−1.95
	Base of cerebrum	−0.93%	−0.43%	3.12%	2.26%	3.48%	−8.10

*Pre1 percent changes values (0%) have been omitted. In the rightmost column, we present the ratio of ROI changes following spaceflight (Post3) compared to the launch abort (Pre3). A ratio >1 indicates that spaceflight induced greater ROI volume changes compared to the launch abort. A negative ratio indicates that the ROI volume changes induced by spaceflight were in the opposite direction as those induced by the launch abort. CAL, Crewmember who experienced the Aborted Launch*.

We then compared the ROI volume changes induced by the launch abort relative to baseline (Pre1 to Pre3) to the ROI volume changes induced by spaceflight relative to baseline (Pre1 to Post3). CAL's brain changes following the launch abort were assessed 64 days after the aborted launch to assess brain changes at a comparable point in recovery time following the aborted launch and spaceflight. We computed the ratio of spaceflight vs. launch abort effects according to the following equation:


$$\begin{array}{l} Spaceflight\;vs.\;Launch\;Abort\;Change\;=\frac{PercentChange_{Post3}}{PercentChange_{Pre3}} \end{array}$$

This ratio thus quantifies the magnitude of CAL's intracranial fluids changes induced by spaceflight vs. the aborted launch. A ratio >1 indicates that spaceflight resulted in fluid changes that were greater in magnitude (i.e., absolute value) compared to those following the launch abort. A ratio <1 indicates the opposite. A positive ratio indicates that the fluid shifts following spaceflight and the launch abort were in the same direction whereas a negative ratio indicates that the fluid shifts following spaceflight and the launch abort were in opposite directions.

## Results

We found no evidence of ventricular volume enlargement nor FW shifts following CAL's aborted launch. Specifically, the slopes of CAL's pre-flight ventricular and FW fractional volume changes were within the variance of pre-flight changes of the control group of ground-based astronauts. Consistent with previous spaceflight studies, upon return from a long-duration ISS mission, CAL exhibited enlargement of the lateral and third ventricles, FW fractional volume decreases at the top of the brain, and FW fractional volume increases around the base. Intracranial fluid changes following the launch abort (Pre3) were smaller and in the opposite direction to post-flight changes measured after a similar post-flight recovery period (Post3).

We quantified the volume of the lateral and third ventricles at each pre-flight session (Pre1-3) in native space using CAT12 in SPM12. We assessed the FW fractional volume within ROIs at the top and base of the cerebrum in native space using a custom FW algorithm (11). We computed CAL's slope of change from before to after the aborted launch as shown in Figures 2A,D, 3A). We then computed Z scores to compare CAL's pre-flight brain changes to brain changes in a control group of 12 ground-based astronauts who underwent two MRI scans pre-flight (Figures 2B,E, 3B).

The slope of CAL's lateral ventricle volume changes from before to after the aborted launch were comparable to those of the control group's pre-flight changes (Figure 2B), with Z scores of 0.49 and 0.24 for the left and right lateral ventricles, respectively. CAL's third ventricle showed a consistent pattern pre-flight with a Z score of 0.68 (Figure 2E). FW analyses yielded similar results such that the slopes of CAL's pre-flight FW shifts at the top and base of the cerebrum were comparable to those of the control group pre-flight (Figure 3C) with Z scores of 0.44 and −0.31, respectively.

We performed a within-subject analysis to compare CAL's intracranial fluid changes following the aborted launch to those following his subsequent spaceflight. CAL's post-flight brain changes were assessed at Post3 to allow for similar recovery periods following the launch abort and spaceflight (i.e., 64 days post-launch abort and 97 days post-flight). CAL's left and right lateral ventricle volume changes following the aborted launch were in the opposite direction and approximately 27 and 5 times smaller, respectively, than post-flight fluid shifts measured at Post3 (Figure 2C; Table 2). Similarly, CAL's third ventricle volume changes following the aborted launch were in the opposite direction and were over 5 times smaller than post-flight expansion (Figure 2F; Table 2). CAL's FW changes at the top and base of the cerebrum following the aborted launch were in the opposite direction and ~2 and 8 times smaller, respectively, than post-flight fluid shifts measured at Post3 (Figure 3C; Table 2).

## Discussion

Here we showed that exposure to hypergravity during launch and landing did not induce lasting intracranial fluid shifts as previously reported with spaceflight. Following the launch abort, CAL's ventricular and FW changes were within range of those changes observed in the ground-based control group. Moreover, CAL's fluid shifts after the launch abort were smaller and in the opposite direction compared to CAL's own brain changes following a subsequent 6-month spaceflight mission.

Spaceflight encompasses exposure to multiple hazards including to radiation and microgravity, confinement to a closed environment with elevated ambient carbon dioxide, social isolation, a heavy workload, disrupted sleep and circadian rhythm, among other factors (23, 24). To our knowledge, these are the only neuroimaging data collected from a crewmember involved in an aborted launch. These data thus offer unique evidence ruling out hypergravity during launch and landing as a contributing factor to previously reported long-lasting intracranial fluid changes following spaceflight. Persisting spaceflight-induced intracranial fluid changes are therefore most likely due to prolonged microgravity exposure and/or other spaceflight factors (23, 24).

## Data Availability Statement

MRI files for this study will be placed in the NASA data repository upon study completion.

## Ethics Statement

The studies involving human participants were reviewed and approved by the Institutional Review Boards at the University of Michigan, the University of Florida, and NASA Johnson Space Center. The patients/participants provided their written informed consent to participate in this study. Written informed consent was obtained from the individual(s) for the publication of any potentially identifiable images or data included in this article.

## Author Contributions

HM analyzed diffusion-weighted data, performed analyses, drafted the manuscript, and created figures and tables. KH preprocessed the ventricular volume data. OP designed the FW analysis pipeline and consulted on interpretation of FW data. RS, JB, AM, and SW designed the experiment and secured funding. HM, KH, OP, SW, TH, and RS edited the manuscript. All authors contributed to the article and approved the submitted version.

## Funding

This study was supported by NASA grant #NNX11AR02G awarded to RS, AM, SW, and JB. HM was supported by a NSERC postdoctoral fellowship and a NASA Human Research Program augmentation grant. KH was supported by a National Institute on Aging fellowship 1F99AG068440.

## Conflict of Interest

AM is employed by KBR. The remaining authors declare that the research was conducted in the absence of any commercial or financial relationships that could be construed as a potential conflict of interest.

## Publisher's Note

All claims expressed in this article are solely those of the authors and do not necessarily represent those of their affiliated organizations, or those of the publisher, the editors and the reviewers. Any product that may be evaluated in this article, or claim that may be made by its manufacturer, is not guaranteed or endorsed by the publisher.
